# The Protective Anticancer Effect of Natural Lycopene Supercritical CO_2_ Watermelon Extracts in Adenocarcinoma Lung Cancer Cells

**DOI:** 10.3390/antiox11061150

**Published:** 2022-06-11

**Authors:** Caterina Di Sano, Valentina Lazzara, Miriana Durante, Claudia D’Anna, Angela Bonura, Paola Dino, Carina Gabriela Uasuf, Elisabetta Pace, Marcello Salvatore Lenucci, Andreina Bruno

**Affiliations:** 1 Institute of Translational Pharmacology (IFT), National Research Council (CNR), 90146 Palermo, Italy; caterina.disano@ift.cnr.it (C.D.S.); valy.lazz@gmail.com (V.L.); claudia.danna@ift.cnr.it (C.D.); angela.bonura@ift.cnr.it (A.B.); pdino@fondazionerimed.com (P.D.); cuasuf@gmail.com (C.G.U.); elisabetta.pace@ift.cnr.it (E.P.); 2 Institute of Sciences of Food Production (ISPA), National Research Council (CNR), 73100 Lecce, Italy; miriana.durante@ispa.cnr.it; 3 Department of Biomedicine, Neuroscience and Advanced Diagnostics (B.N.D.), University of Palermo, 90127 Palermo, Italy; 4 Department of Biological and Environmental Sciences and Technologies (DiSTeBA), University of Salento, 73100 Lecce, Italy

**Keywords:** antioxidants, carotenoids, inflammation, lycopene, lycopene from watermelon, lycopene from gấc, lycopene from tomato, synthetic lycopene, lung cancer, supercritical fluid extraction, tocochromanols

## Abstract

Carotenoids may have different effects on cancer and its progression. The safety of carotenoid supplements was evaluated in vitro on human non-small cell lung cancer (NSCLC) adenocarcinoma A549 cells by the administration of three different oleoresins containing lycopene and other lipophilic phytochemicals, such as tocochromanols. The oleoresins, obtained by the supercritical CO_2_ green extraction technology from watermelon (Lyc W), gấc(Lyc G) and tomato (Lyc T) and chlatrated in α-cyclodextrins, were tested in comparison to synthetic lycopene (Lyc S), by cell cycle, Annexin V-FITC/PI, clonogenic test, Mytosox, intracellular ROS, Western Blot for NF-kB and RT-PCR and ELISA for IL-8. The extracts administered at the same lycopene concentration (10 µM) showed conflicting behaviors: Lyc W, with the highest lycopene/tocochromanols ratio, significantly increased cell apoptosis, mitochondrial stress, intracellular ROS, NF-kB and IL-8 expression and significantly decreased cell proliferation, whereas Lyc G and Lyc T significantly increased only cell proliferation. Lyc S treatment was ineffective. The highest amount of lycopene in Lyc W was able to counteract and revert the cell survival effect of tocochromanols supporting the importance of evaluating the lycopene bio-availability and the real effect of antioxidant tocochromanols’ supplementation which may not only have no anticancer benefits but may even increase cancer aggressivity.

## 1. Introduction

Humans and animals are not able to synthesize the carotenoids that they introduce mostly by eating plants and animal-derived compounds: yellow/orange/red fruits and green leafy edible plants and vegetables, as well as seaweeds, fish and eggs are known to be particularly rich in several kind of carotenoids [1]. Carotenoids belong to the most efficient physical and chemical quenchers of singlet oxygen (^1^O_2_) and are potent scavengers of other reactive oxygen species (ROS) [2,3,4]. Carotenoids comprise more than 700 liposoluble pigments produced by bacteria, cyanobacteria, archaea fungi and plants. Of these, only 20 are detectable both in human blood and cells [5], represented for 90% by α- and β-carotene, β-cryptoxanthin, lutein, lycopene and zeaxanthin [2]. The first important review of the effects induced by carotenoids on human health was published in the early 1950s of the last century [6], reporting the first evidence of these natural compounds in physiological and pathological processes, including lung cancer. Since then, several studies were performed on carotenoids until the publication of the two most well-known large-scale randomized trials on cancer prevention, the CARET [7] and the ATBC [8] studies. They were performed against placebo with vitamin A (25,000 IU/day) and β-carotene (30 mg/day) for CARET, and with α-tocopherol (50 mg/day) and β-carotene (20 mg/day) for ATBC, in more than forty thousand subjects altogether. Both studies were prematurely stopped due to clinical evidence of increased lung cancer and total mortality, against the hypothesis that carotenoids were protective instead. These surprising results were attributed to the dichotomous nature of carotenoids that can act, at the same time, such as anti- or pro-oxidant molecules [2,9]. In physiological and pathological conditions, enzymatic endogenous antioxidant systems (i.e., superoxide dismutase, glutathione peroxidase and catalase) and exogenous non-enzymatic factors (i.e., vitamins E and C, coenzyme Q, β-carotene and glutathione), cooperate to maintain the equilibrium between the production and removal of ROS [10,11,12]. Oxidative stress due to ROS overproduction and its detoxification failure is a great risk factor for the most important chronic non-communicable diseases (NCDs), including cardiovascular diseases, diabetes and cancers [13,14]. In lung cancer, cigarette smoke exposure is one of the main causes that leads to chronic airway inflammation and the activation of inflammatory cells, a source of ROS, involved in several steps of cancer progression [15,16]. Lung cancer is commonly divided into two main categories: non-small cell lung cancer (NSCLC) and small cell lung cancer (SCLC), where 80% are NSCLC, further classified into squamous cell carcinoma, adenocarcinoma and large cell carcinoma [17]. According to the World Health Organization, all cancer types, including lung cancer, are leading causes of death worldwide, accounting for nearly 10 million deaths in 2020, with around one-third associated with smoking habits, high body mass index, alcohol abuse, low fruit and vegetable intake and physical inactivity [18]. Therefore, it appears extremely important to prevent the worst scenario simply by eating a sufficient amount of fresh vegetables and fruits, rich in biologically active compounds. Carotenoids may have a role in the onset, progression and dissemination of lung cancer [9,19]. Lycopene (ψ, ψ-carotene) is a tetraterpene hydrocarbon red pigment with the strongest antioxidant effect among all of the carotenoids, synthesized by plants and photosynthetic microorganisms [20]. It was first found in high concentrations in tomato, but it is also present in a few other pink-orange fruits, such as watermelon, red-fleshed papaya, pink grapefruit, gấc fruit and guava [21]. In the ripe fruits of gấc, a perennial cucurbitaceous plant native to East and Southeast Asia, the content of lycopene can reach up to 2200 µg/g fresh weight, about 70-fold higher than ordinary tomato and watermelon varieties [22]. Thanks to its property as a quencher of singlet oxygen and scavenger of free radicals, lycopene can protect against oxidative stress. Meanwhile, lycopene is widely applied across industrial sectors, such as the food, nutraceutical, pharmaceutical and cosmetic industries as a natural antioxidant and anti-aging product [23], consequently, the investigations into its role for human health are of growing interest. Randomized control clinical trials (RCT), epidemiological studies, in vivo and in vitro studies assessed the anti-inflammatory and antioxidant properties of lycopene revealing a role in the reduction of the inflammatory cytokines and ROS production and supporting its anti-cancer and immuno-modulating activities [24,25,26,27]. Lycopene also interferes with cancer cell proliferation by decreasing cancer cell viability and inducing cell apoptosis [28,29,30,31]. Despite RCTs and prospective studies suggesting a protective effect of a high dietary intake of fruits and vegetables against cancer [32,33] and a recent meta-analysis of prospective studies reporting that higher blood concentrations of several carotenoids and retinol are associated with a reduced lung cancer risk [34] the effect of lycopene, together with other bioactive compounds, in lung cancer is still not well defined. Therefore, in this context, the aim of this study was to evaluate an in vitro model, of the human NSCLC cell line A549, the anti-apoptotic role and the effect on inflammation of three different lycopene-rich oleoresins from watermelon (Lyc W), gấc (Lyc G), and tomato (Lyc T) red-ripe fruits containing carotenoids (mainly lycopene), tocochromanols and lipids, encapsulated in α-cyclodextrins (α-CDs).

## 2. Materials and Methods

### 2.1. Oleoresin Extraction and Encapsulation

The oleoresins containing biological lycopene used in this experimental design were extracted with supercritical carbon dioxide (SC-CO_2_) from the freeze-dried flesh of watermelon (Citrullus lanatus (Thunb.) Mansfeld), gấc (*Momordica cochinchinensis* (Lour.) Spreng) and red-ripe tomato (*Solanum lycopersicum* L.) fruits and encapsulated in α-CDs, as previously described [31], to form stable water suspensions and facilitate intracellular delivery through the plasma membrane [35,36]. The macroscopic and microscopic structure of different oleoresins encapsulated into α-CDs were previously investigated, showing satisfactory oil encapsulation efficiency and the formation of compact and hard shells around dispersed oil-droplets (cyclodextrinosomes) rather than simple host–guest interaction [37,38,39]. The obtained oleoresin/CDs complexes, namely Lyc W, from watermelon, Lyc G, from gấc and Lyc T, from tomato, were characterized by a different amount and composition of carotenoids and tocochromanols, with Lyc W showing the highest all-trans-lycopene/tocochromanols ratio [31] (Table 1).

### 2.2. Cell Culture of A549 CCL-185™

Non-small cell lung cancer (NSCLC) adenocarcinoma cell line (A549 CCL-185™) was purchased from American Type Culture Collection (ATCC, Rockville, MD, USA). Cells were grown as adherent monolayers in RPMI 1640 medium, supplemented with 50 U/mL penicillin, 50 mg/mL streptomycin, 2 mM L-glutamine, 1 mM sodium pyruvate, 10 mM N-2-hydroxyethylpiperazine-N9-2ethane sulfonic acid (HEPES) buffer (Gibco/Invitrogen, Carlsbad, CA, USA) and heat-inactivated (56 °C, 30 min) 10% fetal bovine serum (FBS) at 37 °C in humified atmosphere with 5% CO_2_. When reached at 80–90% confluence, the A549 cells were treated for all of the described below methods with Lyc W, Lyc G, Lyc T or with synthetic lycopene (Lyc S) (dissolved in tetrahydrofuran, purchased from Cayman chemicals, item n. 70945, Ann Arbor, MI, USA) in a starting range concentration for lycopene from 0.5 to 10.0 μM for 24, 48 and 72 h. The best results were obtained at a final concentration of lycopene 10 µM and at a time of 72 h for almost all of the experiment procedures. At least three replicates were performed for each experiment.

### 2.3. Cell Cycle

Stimulated cells for 72 h were harvested, washed twice with ice-cold PBS and suspended at 1 × 106 cells/mL in hypotonic fluorochrome solution (0.1% sodium citrate, 0.03% Nonidet P-40 and 50 μg/mL propidium iodide, PI) for 30 min at room temperature (RT), in the dark [40]. Then, the cells were acquired on a FACSCalibur™ flow cytometer (Becton Dickinson, Mountain View, CA, USA), supported by CellQuest acquisition and data analysis software. Based on DNA content, the apoptotic cells were identified as M1 (sub-G1 phase), M2 (G0/G1 cells), M3 (S cells) and M4 (G2/M cells). Data were expressed as percentage of cells.

### 2.4. Determination of Cell Death and Apoptosis

Cell apoptosis was evaluated by redistribution of phosphatidylserine (PS) staining with annexin V-fluorescein isothiocyanate (FITC) and PI using a commercial kit (Bender Med System, Vienna, Austria), according to the protocol and as previously described [41]. Briefly, treated cells were collected after stimulation for 72 h and, after washing in ice-cold PBS, cells were suspended in 400 μL of binding buffer containing Annexin V-FITC. After incubation for 15 min in the dark at RT, PI was added just before flow cytometry analysis. Cells were analyzed by FACSCalibur™ flow cytometer. The PI negative and PS negative cells, double negative cells (i.e., viable cells) were located in the lower left (LL) quadrant; the PI positive and PS negative cells (i.e., necrotic cells) were located in the upper left (UL) quadrant; the PI positive and PS positive cells, double positive cells (i.e., late apoptotic cells) were located in the upper right (UR) quadrant and the PI negative and PS positive cells (i.e., early apoptotic cells) were located in the lower right (LR) quadrant. Data were expressed as percentage of positive cells.

### 2.5. Analysis of Intracellular Reactive Oxygen Species (ROS)

Intracellular ROS were measured, as previously described [41]. Briefly, after treatment (72 h) with lycopene extracts, the cells were harvested, washed with cold PBS, and stained with 0.5 μM DCFH-DA (30 min, RT in the dark). The esterase activity of the A549 cells was monitored in the presence of peroxides that convert the non-fluorescent DCFH-DA into a highly fluorescent compound, DCF, detected by a FACSCalibur™ flow cytometer. Data were expressed as percentage of DCF positive cells.

### 2.6. Evaluation of Mitochondrial Stress

Evaluation of mitochondrial stress was assessed by the MitoSOX Kit (Molecular Pro-bes Waltham, MA, USA), as previously described [42]. The A549 cells were harvested after 3 h stimulation as required by the MitoSOX kit [40], washed with cold PBS, and incubated with 3 μM MitoSOX reagent at 37 °C in the dark for 15 min. The A549 cells were than washed twice in PBS 1% FBS and analyzed by a FACSCalibur™ flow cytometer. Data were expressed as percentage of positive cells for mitochondrial stress.

### 2.7. Clonogenic Assay

After treatment (72 h) with lycopene extracts, the A549 cells were harvested and seeded out in a six well plate in appropriate dilutions (clonogenic density of 50 cells/cm^2^) to form colonies in 1–3 weeks [43]. During the incubation time for colony formation, cells were maintained in fresh medium at 37 °C in an atmosphere containing 5% CO_2_. At the end of incubation, colonies composed of a minimum of 50 cells, were fixed in 100% methanol and stained with 0.5% crystal violet in 20% methanol. Therefore, the plates were air-dried. The colonies were photographed using a digital camera and counted by using image master 2D count PHICS (count and Plot Histograms of Colony Size) software, a macro written for ImageJ [44].

### 2.8. Western Blot

Western blot analysis for the evaluation of the transcription of nuclear factor kappa B (NF-kB) was performed. The A549 cells were harvested after 3 h stimulation to assess the nuclear expression of NF-kB, as we previously assessed [42], washed with cold PBS, and the cytoplasmic and nuclear protein fractions were separated by a commercial kit “NE-PER Nuclear and Cytoplasmic Extraction Reagents”, following the manufacturer’s directions (Thermo Scientific, Waltham, MA, USA). An amount of 30 μg of total proteins was subjected to sodium dodecyl sulfate-polyacrylamide gel electrophoresis (SDS-PAGE) on 10% gradient gel (Novex) and blotted onto a nitrocellulose membrane. This was blocked with 5% *w*/*v* non-fat dry milk, 1X TBS, 0.1% Tween-20 for 1 h and then probed with a polyclonal rabbit antibody diluted 1:200 in milk, directed against p65/NF-kB (C-20: sc-372, Santa Cruz Biotechnology, Dallas, Texas, USA) and incubated at 4 °C overnight, with gentle shaking. Revelation was performed with a chemiluminescence system (Pierce ECL Western Blotting Substrate, Thermo Fisher, Waltham, Massachusetts, USA), followed by autoradiography applied to detect the signal. Lamin B1 (A-11: sc-377000, Santa Cruz Biotechnology, Dallas, Texas, USA diluted 1:500) was used as a nuclear housekeeping protein to standardize differences in protein loading. Gel images were taken with an EPSON GT-6000 scanner, densitometric analysis was performed using the ImageJ program (the National Institutes of Health).

### 2.9. Real Time PCR

Total RNA was extracted after 24 h of cell stimulation to detect mRNA [15], with TRIzol Reagent (Invitrogen), following the manufacturer’s instructions, and reverse transcribed into first-strand complementary DNA (cDNA), using Moloney murine leukemia virus-reverse transcriptase (M-MLV-RT) and oligo (dT)12–18 primer (Invitrogen). Quantitative real-time PCR of the IL-8 transcript was carried out on Step One Plus Real-time PCR System (Applied Biosystems, Foster City, CA, USA) using specific probe and primers (TaqManGene expression assay for IL-8, Hs00174103m1; Applied Biosystems). The IL-8 gene expression was normalized to glyceraldehyde-3-phosphate dehydrogenase (GAPDH) endogenous control gene (pre-validated TaqMan Gene expression assay for GAPDH, Hs03929097g1; Applied Biosystems, Waltham, Massachusetts, USA) [45]. Relative quantification of gene expression was carried out with the comparative CT method (2−ΔΔCt).

### 2.10. Interleukin-8 (IL-8) Detection Assessment

The release of IL-8 was evaluated by the enzyme-linked immunosorbent assays (ELISA) method (Duo Set R&D Systems, Minneapolis, MN, USA), according to the manufacturer’s instructions. The A549 cell supernatants were recovered after 72 h of stimulation, centrifuged for 15 min at 15,000 g and stored at −80 °C until assayed. At the end of the protocol, the optical density of each well was determined immediately, using a microplate reader set to 450 nm (Microplate reader Wallac Victor2 1420 Multilabel Counter, Perkin Elmer, Waltham, MA, USA).

### 2.11. Statistical Analysis

Analysis of variance (ANOVA) was applied for testing differences between means. The possible association between categorical variables was evaluated by the accurate Fisher’s exact test. A *p*-value < 0.05 was considered statistically significant.

## 3. Results

### 3.1. Lyc W Induces Cell Apoptosis

**Figure 1:**
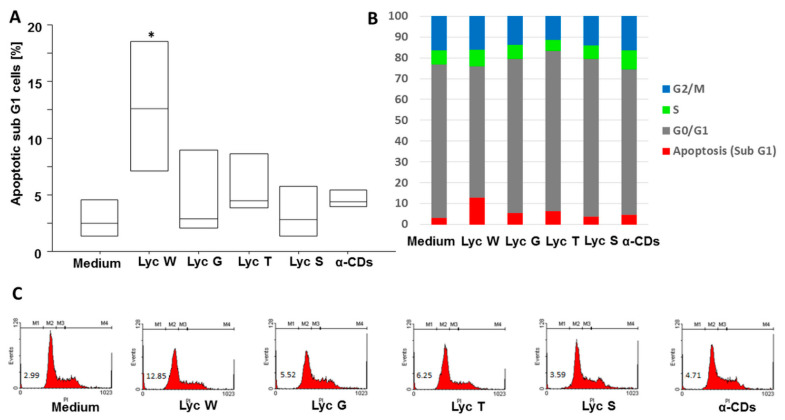
Flow cytometry analysis on A549 cells for cell cycle. (**A**) Lyc W significantly increased the apoptotic cells in comparison to medium, Lyc G, Lyc T, Lyc S and to its carrier α-CDs. The results are shown as box-plots with medians (lines inside the boxes). Analysis of variance (ANOVA), Fisher’s PLSD, *n* = four experiments; *, *p*-value < 0.05 (**B**) Representative graph bar with means for M1, Apoptosis (Sub G1), M2, G0/G1, M3, S, M4, G2/M; (**C**) Representative examples of flow cytometric analysis. The numbers indicate the percentage of positive cells.

**Figure 2:**
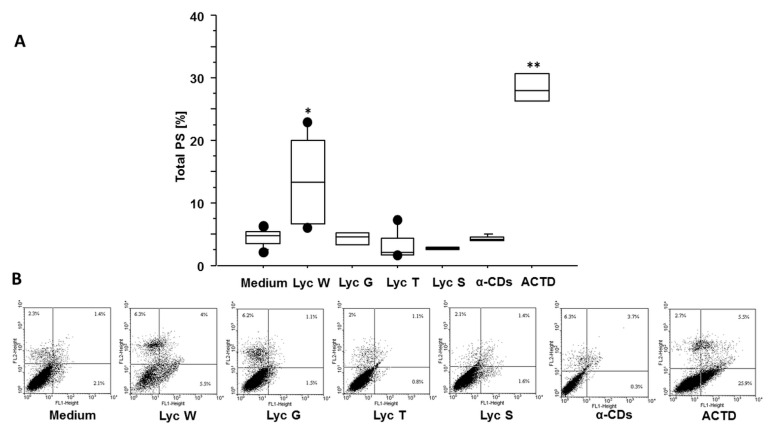
Flow cytometry analysis on A549 cells for Annexin V test, redistribution of phosphatidylserine (PS). (**A**) Lyc W significantly increased total apoptosis in A549 cells in comparison to medium, Lyc G, Lyc T, Lyc S, its carrier α-CDs and to ACTD. The results are shown as box-plots with medians (lines inside the boxes). Analysis of variance (ANOVA), Fisher’s PLSD, *n* = four experiments; *, *p*-value < 0.05, **, *p*-value < 0.01 (**B**) Representative examples of flow cytometric analysis for percentage of positive cells: the numbers indicate the percentage of positive cells for PS, both early and late apoptosis (LR + UR quadrants). FL1-heights, Annexin V; FL2-heights, PI.

We previously reported that lycopene-rich oleoresin/CD complexes influence the A549 cells’ proliferation by MTS test [31], here we wanted also to assess the apoptotic phase with cell cycle and Annexin V test. In the cell cycle, we found that Lyc W significantly increased the apoptotic cells M1 (sub-G1 phase) in comparison with the medium (*p* = 0.0052) and with Lyc G, Lyc T and Lyc S (*p* = 0.0297, *p* = 0.0476 and *p* = 0.0080, respectively) treatments. A significant difference was also observed between Lyc W and its carrier α-CDs (*p* = 0.0172). Lyc S treatment was ineffective (Figure 1A–C). We next investigated whether the pro-apoptotic effect of Lyc W was mediated by the PS redistribution, using the Annexin V test. Lyc W significantly increased total apoptosis, both early and late apoptosis (LR + UR quadrants), in the A549 cells in comparison to the medium (*p* < 0.0001), also in comparison to Lyc G, Lyc T and Lyc S (*p* = 0.0001, *p* < 0.0001 and *p* < 0.0001, respectively). A significant difference was also observed between Lyc W and its carrier α-CDs (*p* < 0.0001), and with Actinomycin D (ACTD) (*p* < 0.0001) as positive control, by inducing apoptosis. At the same time, ACTD also induced a significant increase of apoptosis in comparison with the medium, Lyc G, Lyc T, Lyc S and with the carrier α-CDs (*p* < 0.0001 for all conditions). Again, Lyc S treatment was ineffective (Figure 2A–C).

### 3.2. Effect of All Three of the α-CD Encapsulated Oleoresins on Colony Formation Ability in A549 Cell Line

**Figure 3:**
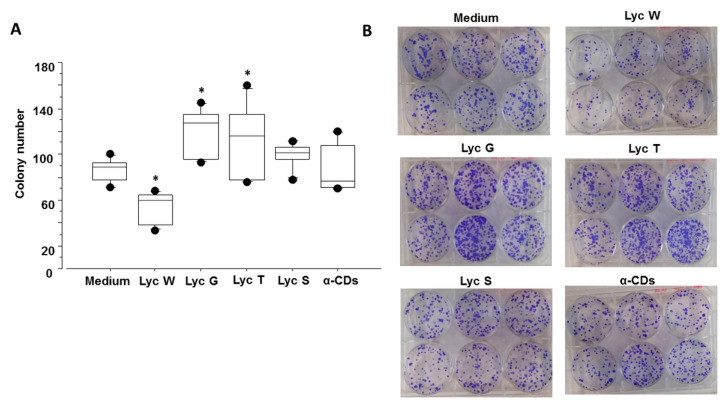
Cell colony assay. Effect of all of the three α-CDs oleoresin extracts on colony formation ability in A549 cell line. (**A**) Lyc W significantly reduced colony numbers whereas Lyc G and Lyc T significantly increased colony numbers when compared to untreated cells. Lyc S treatment was ineffective. The results are shown as box plots with medians (lines inside the boxes). Analysis of variance (ANOVA), Fisher’s PLSD, *n* = six experiments; *, *p*-value < 0.05. (**B**) Bright-field pictures of A549 cell colony are reported.

Lyc W significantly reduced colony numbers when compared to untreated cells (*p* = 0.0098) or to the other experimental conditions, Lyc G, Lyc T and Lyc S (*p* < 0.0001, *p* < 0.0001 and *p* = 0.0006, respectively). In the tested conditions Lyc G, Lyc T but not Lyc S, also had an effect as they significantly increased colony formation compared to the medium (*p* = 0.0072 and *p* = 0.0291, respectively). Furthermore, the effect of all of the CD encapsulated oleoresins was significantly different from that caused by cell stimulation with the carrier α-CDs (*p* = 0.0091, *p* = 0.0077 and *p* = 0.0310 for Lyc W, Lyc G and Lyc T, respectively). Lyc S treatment was ineffective (Figure 3A,B).

### 3.3. Lyc W Effect on Mitochondrial Stress in A549 Cell Line

**Figure 4:**
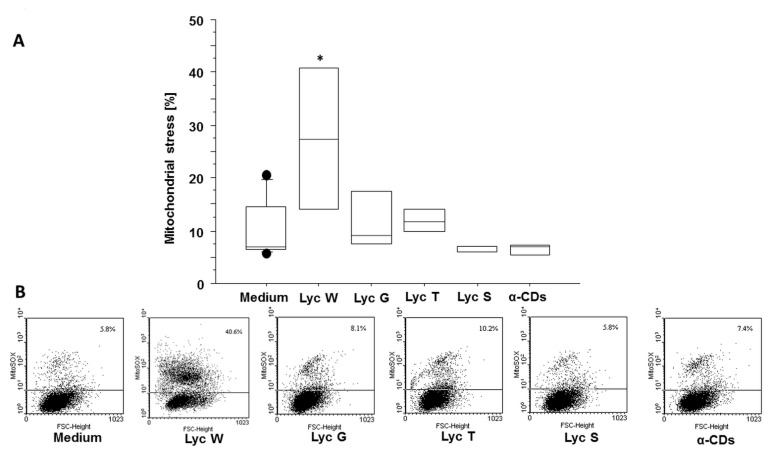
Lyc W effect on mitochondrial stress. (**A**) Lyc W significantly increased mitochondrial stress when compared to cells grown in medium or in Lyc G, Lyc T and Lyc S or in its carrier α-CDs. The results are shown as box-plots with medians (lines inside the boxes). Analysis of variance (ANOVA), Fisher’s PLSD, *n* = five experiments; *, *p*-value < 0.05. (**B**) representative examples of flow cytometric analysis for percentage of positive cells. The numbers indicate the percentage of positive cells for MITOSOX.

It is well known that mitochondrial homeostasis plays a critical role in cancer progression [46]. Based on this background, our results confirmed Lyc W’s protective role on adenocarcinoma cells’ progression as it significantly increased mitochondrial stress when compared to the medium grown cells (*p* = 0.0036) or those incubated in the presence of Lyc G, Lyc T, Lyc S (*p* = 0.0161, *p* = 0.0129 and *p* = 0.0030, respectively) or α-CDs (*p* = 0.0029) (Figure 4A,B).

### 3.4. Lyc W Effect on Spontaneous ROS Generation

**Figure 5:**
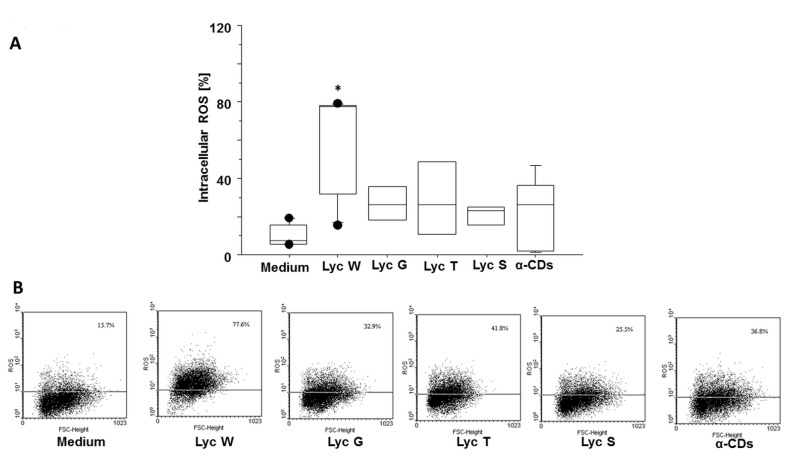
Lyc W affect intracellular ROS generation. (**A**) Lyc W significantly increased intracellular ROS when compared to cells grown in medium or in Lyc G, Lyc T and Lyc S or in its carrier α-CDs. The results are shown as box-plots with medians (lines inside the boxes). Analysis of variance (ANOVA), Fisher’s PLSD, *n* = five experiments; *, *p*-value < 0.05. (**B**) representative examples of flow cytometric analysis for percentage of positive cells. The numbers indicate the percentage of positive cells for DCF.

Besides antioxidant properties, lycopene and other carotenoids may behave as pro-oxidants generating free radical species and ROS that may play a role in cell proliferation and apoptotic cancerous cell death [47]. In confirmation of this, we found that Lyc W significantly increased the spontaneous release of ROS (*p* = 0.0001) also in comparison to cells treated with Lyc G, Lyc T and Lyc S (*p* = 0.0140, *p* = 0.0230 and *p* = 0.0082, respectively) or with its carrier α-CDs (*p* = 0.0029) (Figure 5A,B).

### 3.5. Lyc W Acts on A549 Survival via Enhanced of the Transcription Factor NF-kB and of Pro-Inflammatory Cytokine IL-8

**Figure 6:**
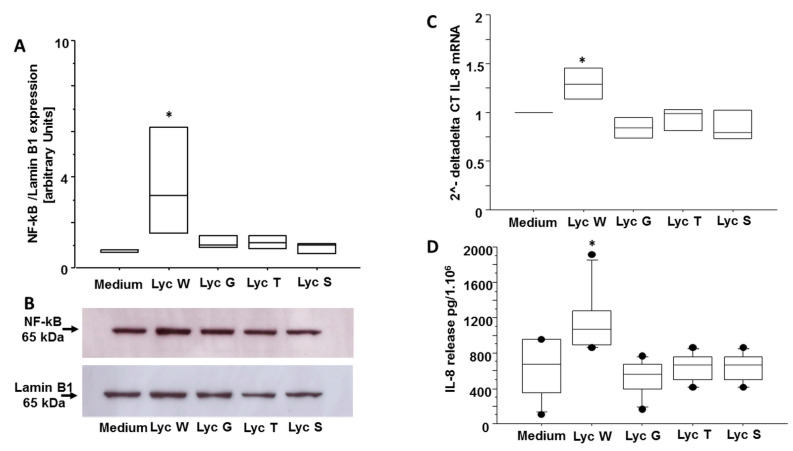
Lyc W affects inflammation markers in A549 cell line. (**A**,**B**) Lyc W significantly increased nuclear translocation of NF-kB expression in comparison to cells treated with medium and in comparison, to cells treated with Lyc G, Lyc T and Lyc S; (**A**) Three Western blots were semi-quantified by densitometric scanning, normalized and expressed after correction with the density of the bands obtained for housekeeping proteins for nuclear proteins. Data are expressed as arbitrary units. The results are shown as box-plots with medians (lines inside the boxes). Analysis of variance (ANOVA), Fisher’s PLSD, *n* = three experiments; *, *p*-value < 0.05. (**B**) representative Western blot for NF-kB and for the nuclear housekeeping gene Lamin B1; (**C**,**D**) Lyc W significantly increased both m-RNA (**C**) and IL-8 protein expression (**D**) in comparison to cells treated with medium and in comparison, to cells treated with Lyc G, Lyc T and Lyc S. The results are shown as box-plots with medians (lines inside the boxes). Analysis of variance (ANOVA), Fisher’s PLSD; *, *p*-value < 0.05. (**C**) *n* = three experiments; quantitative real-time PCR of IL-8; (**D**) *n* = six experiments, ELISA for IL-8 release.

It is widely recognized that cancer and inflammation are deeply related to unhealthy diet regimes [48] and that both NF-kB andIL-8 are correlated with inflammation and the apoptotic pathway in cancer condition [49,50]. Based on these previous evidences, we aimed to assess if Lyc W influence both NF-kB and IL-8 expression. We found that Lyc W significantly increased the nuclear expression of NF-kB in comparison to medium (*p* = 0.0289) and to Lyc G, Lyc T and Lyc S treatments (*p* = 0.0493, *p* = 0.0493 and *p* = 0.0332, respectively) (Figure 6A,B). Likewise, Lyc W significantly increased both IL-8 gene and protein expression in the A549 cells compared to the medium (*p* = 0.0491 for m-RNA and *p* = 0.0016 for protein) and to Lyc G, Lyc T and Lyc S treatments (*p* = 0.0065, *p* = 0.0190 and *p* = 0.0088 for m-RNA and *p* = 0.0003, *p* = 0.0022 and *p* = 0.0022 for protein, respectively) (Figure 6C,D).

## 4. Discussion

Antioxidants, and carotenoids specifically, may have a role in cancer onset and progression. In a previous study we described a method to obtain lycopene-rich oleoresins from watermelon, gấc and tomato fruits by SC-CO_2_ extraction and efficiently encapsulate them into α-CDs [31]. SC-CO_2_ technology represents a green alternative to the conventional techniques for the extraction of lipophilic compounds, typically using harmful organic solvents, thought to improve the biological activity of lycopene and to enhance its bio-availability compared to the intake of fresh or processed lycopene-containing foods [51,52,53,54]. The oleoresin/α-CD complexes, containing, in addition to lycopene, the entire lipophilic phyto-complex of the fruits from which they were extracted (i.e., other carotenoids, tocochromanols and unsaturated fatty acids), demonstrated high antioxidant activity with differential ability in quenching radical cations and scavenging peroxy-radicals. Furthermore, when used in preliminary cytotoxicity tests on human lung adenocarcinoma A549 cell cultures, the oleoresin/α-CD complexes differentially affected cell viability (evaluated by the MTS assay) in relation with their total lycopene/total tocochromanols ratios. Thus, the oleoresin extracted from watermelon encapsulated in α-CDs (Lyc W), the one with the highest ratio value (10.53; Table 1), led to a significantly decreased cell viability [31]. Based on the above mentioned evidence, we further investigated the role of these oleoresin/α-CD complexes (Lyc W, Lyc G and Lyc T) in comparison to synthetic all-trans lycopene (Lyc S) on A549 cell apoptosis, oxidative stress and inflammation. The following new findings are reported: (1) Lyc W significantly increased spontaneous cell death and apoptosis, as well as spontaneous ROS production, mitochondrial stress, the nuclear expression of the transcription factor NF-kB and IL-8 production, whereas it significantly decreased cell proliferation; (2) all of these events were significantly different between Lyc W and Lyc G, Lyc T and Lyc S; (3) Lyc G and Lyc T did not have any effect on cell death and apoptosis as well as on spontaneous ROS production, mitochondrial stress, NF-kB expression and IL-8 production, whereas they significantly increased cell proliferation; (4) Lyc S had no effect on any experimental conditions. Despite the fact that this study is an experimental in vitro design, it provides additional information on the potential role of lycopene in preventing lung cancer progression by counteracting the effect of other antioxidants, such as tocochromanols. Interestingly, we found that synthetic all-trans lycopene had no effect on any of the experiments performed, confirming the importance of using the biological extract of lycopene from fresh fruits and reinforcing the concept related to the importance of lycopene being highly bio-available for its activity in human health: indeed, the Lyc W used in this experimental design was a natural lycopene extracted from fresh fruits and encapsulated in a system that allowed it to fully penetrate the cells. This may be also relevant for the best benefit for human health to consume a safe and efficient supplement derived from the whole phyto-complex, rather than chemically synthesized lycopene. Furthermore, all of the extracts used in this study are oleoresins that represent both a good and a safe model of the synergy between natural biological lycopene with other antioxidant biomolecules with a high lipophilic antioxidant activity. In addition, these oleoresins are an excellent product for experimental testing and design on the cancer disease prevention model in vitro [42]. The interesting results that we found in this study about the opposite role in lung adenocarcinoma A549 cell line of lycopene and of the other bioactives present in the oleoresin/α-CD complexes, such as tocochromanols, is due to the well-proven dichotomous nature of several antioxidants, mainly in the context of carcinogenesis [2,47]. As previously described [31] and here reported in Table 1, the oleoresin/α- CD suspensions used in this experimental design not only featured a different amount of total carotenoids, included all-trans lycopene and tocochromanols, but also a different ratio among all of the antioxidants compounds: Lyc W exhibited both the highest amount of total carotenoids and the highest lycopene/tocochromanols ratio (10.53), followed by Lyc G (6.6) and by Lyc T (0.37) with an amount of total carotenoids of 7509 mg/kg, 4629 mg/kg and 5217 mg/kg, respectively [31]. In our opinion, this strong difference in the lycopene/tocochromanols ratio, in favor of total lycopene for Lyc W extracts, is the main cause for the effect that we observed in our pre-clinical in vitro study on apoptosis, cell proliferation and inflammation of the adenocarcinoma A549 cell line. In addition, previously we also assessed [31] that α-tocopherol, the most biologically active form of vitamin E, was the dominant form heavily represented in Lyc G and Lyc T, whereas it was not detected at all in the Lyc W extract. Natural vitamin E represents a group of compounds, including eight different compounds, four tocopherols and four tocotrienols. It is considered one of the most potent anti-oxidants involved in chronic inflammatory diseases [55]. Actually, the beneficial effects of vitamin E supplementation are still discussed in literature. Controversial results are shown for vitamin E activity, especially in the context of cancer, either in lung cancer [7,8] and in prostate cancer [56,57], where its supplementation appears more as a risk factor for a higher incidence of cancer, rather than a protective compound useful for the prevention or for counteracting cancer progression. Our results are exactly in line with this latter evidence. Cancer is a complex disease that arises from an interplay between genetic and environmental factors. The International Agency for Research on Cancer assessed that the global cancer burden is expected to be 28.4 million cases in 2040, 11% related exclusively to lung cancer. Particularly, NSCLC accounts for significant morbidity and mortality worldwide, unfortunately with most patients diagnosed at advanced stages, with the need to find more appropriate tools of precision medicine in the care of lung cancer. In lung cancer, tobacco smoke is one of the main causes that leads to chronic airway inflammation and activation of inflammatory cells, which are a source of ROS with highly mutagenic molecules that can support the progression to malignancy of tumor cells, as well as cellular proliferation, apoptosis, metastasis and angiogenesis [58]. At the same time, an important role in lung cancer development is also due to inflammation. In this context, both the activation of NF-kB that plays an important role in controlling cell survival and apoptosis and the consequent release of proinflammatory cytokines, such as IL-8, can be considered the bridge between inflammation, cancer and oxidative stress [59,60,61]. It has already been assessed, in the context of chemoprevention, that the redox-sensitive transcription factors NF-κB and IL-8 are inflammatory signaling mediators involved in oxidative stress either in human respiratory epithelial cells or in cancer epidermal alterations [62,63]. A recent study performed both with primary epidermal keratinocytes and in a 3D epidermis model showed that a chemotherapeutic agent, also approved for the treatment of NSCLC, induced an increased rate of oxidative stress, inflammation and apoptosis via the upregulation of both NF-κB and IL-8 expression [62]. Furthermore, other studies assessed that IL-8 expression correlated with ROS production, with ROS-induced mitochondrial damage and with the pro-apoptotic activity of chemopreventive agents, by demonstrating an increased expression of IL-8 in both human breast cancer and neuroblastoma cell [49,50]. Our results are in line with this latter evidence, in identifying Lyc W as a pro-apoptotic factor of the A549 cell line, that increases either the nuclear expression of the redox-sensitive transcription factors NF-κB, or both the m-RNA and protein level of IL-8, further supporting the chemopreventive role that lycopene can have in lung cancer, through its pro-oxidant and pro-inflammatory activity. In this latter condition, carotenoids, and particularly lycopene, can be protective against cancer. As carotenoids, another antioxidant category, such as polyphenols, can exert antioxidant or pro-oxidant cytotoxic effects, by low or high level of ROS production, depending on their endogenous concentration [62]. In this context, another of our previous pre-clinical studies on the effect of antioxidants on lung cancer, performed also with the A549 cell line [41], showed the role played by the flavonoid apigenin and the adipocytokine leptin in cell survival. These were able to abolish and reverse the proliferative and the anti-death roles promoted by the survival adipocytokine, leptin, also in the presence of lung adenocarcinoma pleural fluids promoting cancer growth in the milieu. An important signaling pathway involved in the development and progression of cancer is the oxidative response that modulates the expression of genes implicated in cellular differentiation, proliferation, apoptosis and immune response [64,65,66]. In this prospective, carotenoids’ intake from a healthy diet rich in natural antioxidants, such as colorful fruits and vegetables and their increased plasmatic level, are long since under investigation to assess their protective role against chronic diseases, such as NCDs, lung cancer included. Nevertheless, in vitro, ex vivo and in vivo studies, including interventional trials with isolated carotenoids and antioxidants as supplements, displayed controversial results, often with no benefit for human health or even adverse and harmful consequences [7,8]. We also have to consider that our knowledge of carotenoids and their impact on human and lung health is lacking due to the interaction with several factors that influence the homeostasis of metabolism and of the lung. This experimental study has two main limitations: some markers related to ROS production, such as the nuclear factor-erythroid factor 2-related factor 2 (Nrf2) and glutathione peroxidase (GPx) and related to apoptosis as caspase-3, caspase-9 and Poly (ADP-ribose) polymerase (PARP) were not evaluated. These markers will be under investigation in a future study.

## 5. Conclusions

At present, together with the evidence in literature, our results suggest that all antioxidant compounds, including lycopene and tocochromanols, exert biological effects on lung cancer, by mechanisms affecting the expression of inflammatory markers. We strongly recommend that the antioxidant intakes by supplements should be controlled and taken only if necessary, as they could be detrimental and not healthy or therapeutic, due to their documented impacts on inflammation related to lung cancer. Primary prevention by a healthy style life is the first choice, including following the Mediterranean diet and avoiding the habit of smoking. To strengthen this concept, our in vitro results, strongly supported by the previous pre-clinical evidence, should lead the researchers to better investigate the role of lycopene together with the other carotenoids in well-designed clinical studies, that should also include some important aspects, such as their bio-availability and their strategy for extraction and storage, to identify an effective approach for the primary prevention and for a tailored therapy. In this scenario, the concept of primary prevention is really important, by introducing natural potent antioxidants through a healthy diet rich in whole fruits, cereals and vegetables that maintain a general homeostasis and a strength function of the immune systems, rather than counteracting the effect of the oxygen free radicals with specific supplements that can be harmful by increasing oxidative stress and cancer progression.

## Figures and Tables

**Table 1:** Biochemical composition of Lyc W, Lyc G and Lyc T oleoresin/α-CD suspensions.

	Lyc W	Lyc G	Lyc T
Total carotenoids (μg/mL suspension)	504.5 ± 5.5	89.0 ± 5.4	266.0 ± 17.1
All-trans-lycopene	215.8 ± 1.1	49.1 ± 2.1	152.7 ± 9.2
lycopene cis isomers	20.2 ± 0.9	7.7 ± 1.2	33.6 ± 1.7
α-carotene	27.0 ± 2.0	2.3 ± 0.2	16.0 ± 2.7
β-carotene	241.5 ± 1.5	29.9 ± 1.9	63.7 ± 3.5
Total tocochromanols (μg/mL suspension)	22.4 ± 1.8	8.6 ± 0.9	498.5 ± 16.9
α-tocopherol	nd	8.6 ± 0.9	425.7 ± 12.3
γ-tocopherol	8.9 ± 0.9	nd	72.8 ± 4.6
γ-tocotrienol	13.5 ± 0.9	nd	nd
*Total lycopene/Total tocochromanols*	*10.53*	*6.6*	*0.37*

Biochemical composition of Lyc W, Lyc G and Lyc T oleoresin/α-CD suspensions, modified from Bruno et.al [31]. Values represent the mean ± standard deviation of three independent replicates (*n* = 3). Italic formatting identifies dimensionless ratios.

## Data Availability

Data is contained within the article.
